# “N‐*π*‐N” Type Oligomeric Acceptor Achieves an OPV Efficiency of 18.19% with Low Energy Loss and Excellent Stability

**DOI:** 10.1002/advs.202202513

**Published:** 2022-06-16

**Authors:** Lili Zhang, Ziqi Zhang, Dan Deng, Huiqiong Zhou, Jianqi Zhang, Zhixiang Wei

**Affiliations:** ^1^ CAS Key Laboratory of Nanosystem and Hierarchical Fabrication National Center for Nanoscience and Technology Beijing 100190 China; ^2^ Sino‐Danish Center for Education and Research Sino‐Danish College University of Chinese Academy of Sciences Beijing 100190 China; ^3^ School of Nanoscience and Technology University of Chinese Academy of Sciences Beijing 100049 China

**Keywords:** energy loss, N‐*π*‐N type, oligomeric acceptor, organic solar cells, thermal stability

## Abstract

A novel “N‐*π*‐N” type oligomeric acceptor of 2BTP‐2F‐T, constructed by two small non‐fullerene acceptor (NFA) units linked with a thiophene *π* bridge is reported. The 2BTP‐2F‐T not only combines the advantages of small NFA and polymeric acceptors (PYF‐T‐*o*) with similar units but also exhibits superior characteristics of high absorption coefficient and high electron moblity(*µ*_e)_) with less dependence on molecular packing. Using PM6 as the donor, a remarkable efficiency of 18.19% is obtained with an open circuit (*V*_oc_) of 0.911 V, short current circuit (*J*_sc_) of 25.50 mA cm^−2^, and fill factor (FF) of 78.3%, which is much better than that of the corresponding monomer (16.54%) and PYF‐T‐*o* (15.8%) based devices. The much‐improved efficiency results from two aspects: 1) an enhanced FF due to the largely improved *µ*_e_ and well‐controlled morphology ; 2) a higher value of (*J*_sc_ × *V*_oc_) due to its higher absorption coefficient and efficient charge generation at a similar low energy loss. Furthermore, the PM6/2BTP‐2F‐T device possesses the longest T_80_ lifetime to light‐soaking and comparable high thermal stability with PM6/PYF‐T‐*o*. The results indicate that the “N‐*π*‐N” type oligomeric acceptor has a great application prospect due to its superior high efficiency and improved stability in organic solar cells.

## Graphical abstract

A novel oligomeric acceptor 2BTP‐2F‐T, constructed by linking two non‐fullerene acceptors with thiophene, is reported. It exhibits superior characteristics over its monomer and polymer, including higher absorption coefficient and electron mobility in pristine materials, and welltuned morphology in PM6 blends, pushing an attain of efficiency of 18.19% with improved fill factor and short circuit current under similar energy loss.

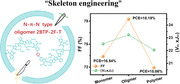

## 1 Introduction

Organic solar cells (OSCs) show excellent application prospects in flexible and portable devices because of their light‐weight property, flexibility, and low cost.^[^ ^1^, ^2^, ^3^, ^4^, ^5^, ^6^, ^7^ ^]^ The polymer‐donor‐based OSCs, according to the compositions of the active layer, fall into two categories: polymer‐small molecule acceptor and all‐polymer OSCs.^[^ ^8^, ^9^, ^10^, ^11^ ^]^ The invention of small molecular non‐fullerene acceptors (NFA), such as ITIC, Y6, and their analogs, has pushed the OSC efficiency to higher than 18%, and all‐polymer OSCs efficiency over 17% with high thermal stability.^[^ ^2^, ^3^, ^10^, ^12^, ^13^, ^14^ ^]^ An oligomer‐type acceptor with higher molecular weight (MW) would be expected to combine the advantages of both, because 1) compared to its small NFA counterpart, it is anticipated to decrease the diffusion rate in the blend films by the vastly increased MW, improving the device stability;^[^ ^15^ ^]^ 2) compared to its polymeric counterparts, it could obtain definite molecular structure, absence of chains entanglements and increased diffusion coefficient,^[^ ^15^, ^16^ ^]^ leading to an easier morphology control and improved device performances.

Then, the main point is how to construct an oligomeric acceptor to achieve the advantages mentioned above. The traditional oligomers feature identical structures with definite repeating units, and their MW should be located between 1000 of the “small molecules” and 10 000 of the polymers.^[^ ^17^ ^]^ Currently, small molecules of typical NFA and small molecular donors sometimes are also called oligomeric molecules with MWs of ≈1500–2000. Obviously, the MW of oligomers with less than 2500 can hardly meet our requirements. Oligomeric conjugated molecules with three or more definite arms are also reported. However, their MW is still hardly larger than 2500, due to their small repeating units such as the perylene diimine, naphthalene diimide, diketopyrrolopyrrole, or their derivatives; additionally, their poorer absorption spectrum or non‐ideal charge transport resulted from their non‐planar molecular backbone or non‐ideal morphology limit their device performances.^[^ ^17^, ^18^, ^19^ ^]^ That is a similar situation for ladder‐core armed with electron‐deficient end groups.^[^ ^20^, ^21^, ^22^ ^]^ Motivated by the excellent device performance of the polymer acceptor with a moderate MW (≈20 000),^[^ ^20^, ^23^ ^]^ and also the rigid molecular backbone of Y‐series NFA, linking the efficient Y‐series NFA in a proper sequence might be a possible strategy to construct our objective oligomer‐type acceptors.

**Figure 1:**
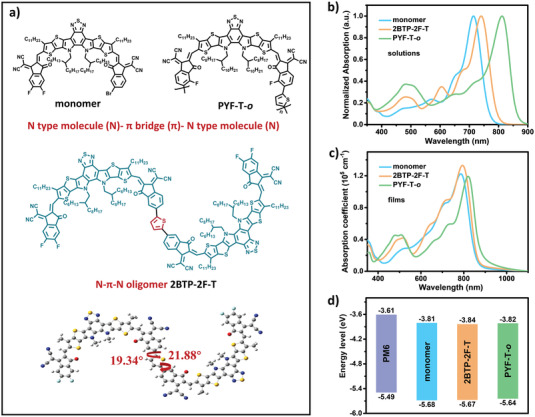
Molecular properties: a) molecular structure; b,c) absorption spectrum and absorption coefficient for the three acceptors in film; d) energy levels calculated from SWV.

In this article, we designed and successfully synthesized an “N‐*π*‐N” type of oligomeric acceptor **2BTP‐2F‐T** (**Figure** **1**a) with a high MW (3359) through linking two efficient Y‐series NFA molecules (N type) by the thiophene *π* bridge. Compared to corresponding monomer and polymeric acceptor (PYF‐T‐*o*) with similar building units, the N‐*π*‐N oligomeric acceptor **2BTP‐2F‐T** exhibits not only a combined advantage of them, but also superior molecular properties, including an in‐between absorption range but with a higher absorption coefficient, and enhanced electron mobility with moderate molecular packing. Using PM6 as the donor, **2BTP‐2F‐T** obtained a remarkable power conversion efficiency (PCE) of 18.19% with an open‐circuit voltage (*V*_oc_) of 0.911 V, a short‐circuit current (*J*_sc_) of 25.50 mA cm^−2^, and a fill factor (FF) of 78.3%, which is much higher than that of the corresponding monomer (16.5%) and PYF‐T‐*o* (15.9%) based devices. The excellent device performance for PM6/**2BTP‐2F‐T** is a combined result of improved FF and enhanced value of (*J*_sc_ × *V*_oc_) with a small energy loss (0.53 eV), due to the joint contributions of its excellent material property, and easily well‐tuned morphology with a small Urbach energy of 22.74 meV. Additionally, the encapsulated PM6/**2BTP‐2F‐T** device possesses the longest T_80_ lifetime (7 times longer than PM6/monomer, and 2.4 times longer than PM6/polymer) under illumination in the air condition, and its thermal stability is also comparable to that of PM6/ PYF‐T‐*o*. The result indicates that the “N‐*π*‐N” type oligomeric acceptor has promising application due to its ability to obtain maximized efficiency and improved stability in OSCs.

## 2 Results and Discussion

### 2.1 Material Design and Molecular Properties of **2BTP‐2F‐T**

The monomer was synthesized through an asymmetrical Knoevenagel reaction from its precursor BTP‐CHO (Scheme S1, Supporting Information). To improve the yield of the reported asymmetrical Knoevenagel reaction, we adopted a two‐step heating method (reacted at room temperature for 3 h, then heated to 65 °C overnight) to ensure the selectivity of the reaction, and the yield was slightly improved to 41.5%.^[^ ^24^, ^25^ ^]^ The oligomeric acceptor **2BTP‐2F‐T** was prepared through Stille coupling with monomer and linker with a high yield of 70%. Consequently, compared to the symmetrical NFA or the monomer, the synthesis of **2BTP‐2F‐T** only takes an extra step but with a high yield; in contrast to the polymer (PYF‐T‐*o*), the yield of **2BTP‐2F‐T** was probably higher due to its easier purification, no MW distribution, and no batch‐to‐batch variations. Noteworthy, because the performance of commercial PJ1^[^ ^23^ ^]^ is not as good as PYF‐T‐*o*,^[^ ^7^ ^]^ we use PYF‐T‐*o* as its corresponding control polymer.

As seen in the top view of **2BTP‐2F**−T (Figure 1a), the dihedral angle between the linker and monomer is ≈20°, which is decreased by ≈15° than the compound without the linker (Figure S1, Supporting Information). This result demonstrates that the thiophene linker well alleviates the crowded space between the two end‐capped acceptors, which leads to a more planar molecular backbone and a longer effective conjugated length. Additionally, **2BTP‐2F**−**T** exhibited good solubility in common solvents, including chloroform (CF), tetrahydrofuran (THF), and chlorobenzene (CB), demonstrating its good solution processability.

The UV–vis absorption spectra of **2BTP‐2F‐T**, monomer, and PYF‐T‐*o* were investigated and compared both in the CF solutions and neat films, and the detailed data are summarized in Table S1, Supporting Information. As shown in Figure 1b, there is a big disparity in their absorption spectrums for the three molecules in the CF solution. Compared to **2BTP‐2F‐T**, PYF‐T‐*o* exhibits the red‐shifted absorption while the monomer shows the blue‐shifted absorption spectrum, which should be due to easier pre‐aggregation for the polymer and the increased effective length for the dimer. The tendency of their absorption‐onset variation in the film was consistent with their solution; however, their differences become smaller, which is ascribed to the largest red‐shifts for the monomer and the least red‐shifts for the polymer (Figure 1c). The variations in the solution and film differences result from their different assemble abilities, which will be discussed in the following. The absorption shape of **2BTP‐2F‐T** is more similar to the monomer than that of PYF‐T‐*o*, indicating that **2BTP‐2F‐T** retains the absorption properties of the monomer. Importantly, **2BTP‐2F‐T** presents the highest photon absorption capacity due to its most broadened absorption spectrum from 600 to 900 nm and its highest absorption coefficient (1.33 × 10^5^ cm^−1^) at the maximized absorption peak. The superior absorption for **2BTP‐2F‐T** should be the combined effect of its higher absorption coefficient of dimer in solution due to its increased molecular conjugation (Figure S2, Supporting Information) and well‐assemble ability, indicating a probable high *J*_sc_ for the devices. Noteworthy, although PYF‐T‐*o* exhibits a slightly red‐shifted absorption, it exhibits a much lower absorption coefficient in the range of 600–800 nm than that of monomer and **2BTP‐2F‐T**.

The square‐wave voltammetry (SWV; Figure S3, Supporting Information) was carried out to measure their electrochemical properties, and their frontier energy levels are aligned in Figure 1d. The frontier energy levels were calculated from the onset oxidation (*E*_OX_) and reduction (*E*_RE_) potentials using the following equations: *E*_HOMO_ = −*e*(*E*_OX_ − *E*_Fc/Fc +_ + 4.8), *E*_LUMO_ = −*e*(*E*_RE_ − *E*_Fc/Fc +_ + 4.8). The calculated highest occupied molecular orbital (HOMO) energy levels were −5.68, −5.67, and −5.64 eV for monomer, **2BTP‐2F‐T**, and PYF‐T‐*o*, respectively. And their corresponding lowest unoccupied molecular orbital (LUMO) energy levels were −3.81, −3.84, and −3.82 eV. In comparison to the monomer, **2BTP‐2F‐T** shows a slightly higher HOMO but lower LUMO energy level, which is consistent with its slightly red‐shifted absorption and indicates a probable decreased *V*_oc_. Summarily, **2BTP‐2F‐T** exhibits similar in‐between energy levels compared with monomer and PYF‐T‐*o*, which is a matched energy level with PM6 (Figure 1d).

**Figure 2:**
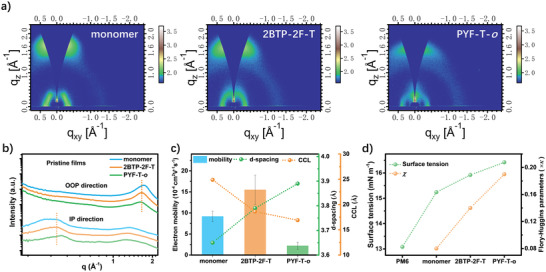
Molecular packing abilities: a) 2D GIWAXS images; b) corresponding 1D curves for GIWAXS; c) electron mobility, d‐spacing, and CCL in *π*–*π* stacking direction; d) surface tension calculated from contact angle and Flory–Huggins interaction parameters between PM6 and the three acceptors.

The grazing incidence wide‐angle X‐ray scattering (GIWAXS) and contact angle measurements were carried out to characterize the molecular packing at thermal annealing (TA) condition (TA = 100 °C), and surface tension of three materials. As shown in their 2D images and 1D curves (**Figure** **2**a,b), the **2BTP‐2F‐T** adopts a preferential face‐on packing mode, the same as that of the PYF‐T‐*o* and monomer, illustrated from their prominent (010) peak in out‐of‐plane (OOP) direction and (100) peak in‐plane (IP) direction. The calculated *π*–*π* stacking distance for (010) peak in the OOP direction is 3.64, 3.79, and 3.87 Å, for monomer, **2BTP‐2F‐T,** and PYF‐T‐*o*, respectively; their corresponding coherence crystal length (CCL) is 24.9, 18.6, and 16.9 Å, respectively. The d‐spacings and CCLs of **2BTP‐2F‐T** are located in the middle of monomer and PYF‐T‐*o*, indicating its in‐between molecular assemble ability, which well explains its intervening absorption red‐shifts from solution to film as we discussed above. This inferior molecular packing of **2BTP‐2F‐T** than that of monomer is probably due to its slightly un‐planar molecular backbone resulted from the joint of the two monomers; its more ordered packing than that of polymer should be attributed to its much weaker or absence of molecular chain entanglement, which could be observed from the increased *q* value of the (100) peak (Figure 2b).

Out of our expectation, although **2BTP‐2F‐T** exhibits moderate molecular packing ability and *π*–*π* stacking distance, however, it obtains the highest averaged electron mobility (*μ*_e_) of 1.55 × 10^−3^ cm^2^ V^−1^ s^−1^, which is slightly higher than that of the monomer (9.17 × 10^−4^ cm^2^ V^−1^ s^−1^) and much higher than that of PYF‐T‐*o* (2.20 × 10^−4^ cm^2^ V^−1^ s^−1^) (Figure 2c). Compared to the *µ*_e_ of the monomer, the higher value for **2BTP‐2F‐T** with less molecular packing ability is probably due to its more contribution of charge transport in intra‐molecules, resulting from its double prolonged molecular backbone in **2BTP‐2F‐T**.^[^ ^26^, ^27^ ^]^ Importantly, the *µ*_e_ with less dependence on molecular packing ability indicates that the N‐*π*‐N type oligomeric acceptor owns a better tolerance to morphology, which is a prominent advantage over its monomer and polymer due to the morphology control being a tough‐nut in all currently reported type of OSCs.

The change of the molecular backbone would also impact the molecular surface tensions and lead to a different Flory–Huggins interaction parameter *χ*, and ultimately act on the morphology. The surface tensions calculated from contact angle (Figure 2d and Figure S4, Supporting Information) were 15.24, 15.92, and 16.42 mN m^−1^ for monomer, **2BTP‐2F‐T**, and PYT‐F‐*o*, respectively, all of which were close to that of PM6 (13.08 mN m^−1^). The in‐between surface tension for **2BTP‐2F‐T** leads to an intervening Flory–Huggins interaction parameter *χ* for PM6/**2BTP‐2F‐T** (*χ *= 0.14), compared to that of PM6/monomer (0.08) and PM6/PYF‐T‐*o* (0.19).

### 2.2 Fabrication and Device Performance of the OSCs

**Figure 3:**
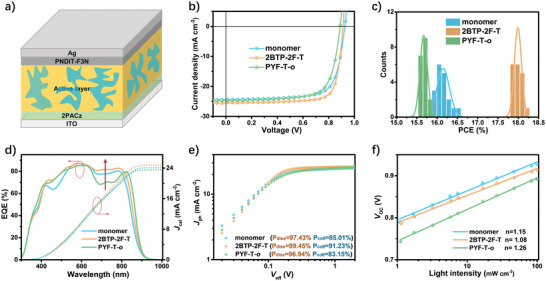
Device performances: a) device structure; b) best *J*–*V* curves; c) statistical distributions of the three PCEs of the best devices (≈20 pieces); d) the EQE curves and calculated *J*_sc_ from EQE based on the best devices; e) relationship between *J*_ph_ and *V*_eff_; f) *V*_oc_ dependence on incident light intensity.

Using PM6 as the donor, a conventional device with a structure of ITO/[2‐(9H‐carbazol‐9‐yl)ethyl]phosphonic acid (2PACz)/active layer/poly[(9,9‐bis(3'‐(N,N‐dimethylamino)propyl)‐2,7‐fluorene)‐alt‐5,5'‐bis(2,2'‐thiophene)‐2,6‐naphthalene‐1,4,5,8‐tetracaboxylic‐N,N'‐di(2‐ethylhexyl)imide] (PNDIT‐F3N)/Ag was fabricated to investigate the device performance of **2BTP‐2F‐T** (**Figure** **3**a). The fabrication conditions were carefully tuned by adjusting the ratios between donor and acceptor, thermal annealing temperatures, concentrations of additives, and different hole/electron transport layers to improve the device performance. The detailed optimization results and conditions are summarized in Tables S2–S4, Supporting Information. Surprisingly, the device based on PM6**/2BTP‐2F‐T** exhibits a superior PCE of 18.19% with a *V*_oc_ of 0.911 V, a *J*_sc_ of 25.50 mA cm^−2^, and an FF of 78.3%, which is much better than that of PM6/monomer and PM6/PYF‐T‐*o*. The best PCE for PM6/monomer is 16.5% with a *V*_oc_ of 0.925 V, a *J*_sc_ of 24.4 mA cm^−2^, and an FF of 73.4%; and the best PCE for PM6/PYF‐T‐*o* was 15.9% with a *V*_oc_ of 0.889 V, a *J*_sc_ of 24.8 mA cm^−2^, and an FF of 71.9%.

The slightly higher PCE of PM6/PYF‐T‐*o* in this study compared to the reported literature should be attributed to the changed electron/hole interlayer.^[^ ^7^ ^]^ The best *J*–*V* curves and statistical distributions of PCEs of ≈20 pieces of devices are shown in Figure 3b,c for the three systems. Compared to the other two, the apparent advantage of PM6**/2BTP‐2F‐T** is its high *J*_sc_ and FF. The tendency for the *V*_oc_ variation has mainly resulted from their differed energy bandgap rather than the energy loss, which we will discuss in the following section. As seen from the external quantum efficiency (EQE) curves of the best devices (Figure 3d), the disparity in *J*_sc_ for the three devices is mainly in the range of 600–800 nm, which is in the absorption range of the acceptor. Hence, the higher *J*_sc_ of PM6**/2BTP‐2F‐T** should be a joint contribution to its higher absorption coefficient and the efficient utilization of the absorbed photons. Noteworthy, the calculated *J*_sc_ from EQE is in good agreement with that measured from the *J*–*V* tests within an error of 5%, and the best photovoltaic parameters are summarized in **Table** **1**.

**Table 1:** Detailed photovoltaic parameters of devices with the architecture of ITO/2PACz/active layer/PNDIT‐F3N/Ag

Active layer	*V*_oc_ [V]	*J*_sc_ [mA cm^−2^]	FF [%]	PCE [%]	*J*_sc_^EQE^ [mA cm^−2^]
PM6: monomer	0.925 (0.923 ± 0.003)	24.35 (24.05 ± 0.22)	73.40 (73.20 ± 0.40)	16.54 (16.25 ± 0.14)	23.52
PM6:2BTP‐2F‐T	0.911 (0.907 ± 0.004)	25.50 (25.58 ± 0.18)	78.28 (77.84 ± 0.44)	18.19 (18.05 ± 0.07)	24.77
PM6: PYF‐T‐o	0.889 (0.889 ± 0.002)	24.80 (24.56 ± 0.23)	71.94 (72.07 ± 0.52)	15.86 (15.74 ± 0.05)	24.08

Average values and standard deviations were obtained from the top ten devices, which were expressed as mean ± SD, *n* = 10.

The efficient utilization of the absorbed photons and high FF in PM6/**2BTP‐2F‐T** was convinced by the photo‐physics characterization. The photocurrent density (*J*_ph_, the current density difference under illumination and in the dark) dependent on the effective voltage (*V*_eff_, the voltage difference between the voltage at *J*_sc_ = 0 and applied bias) (Figure 3e) was applied to evaluate the efficiency of charge generation and collection. At a sufficiently high *V*_eff_, all excitons are hypothesized to separate into free charges and the photo‐generated current reaches the maximum saturation *J*_sat_, and the exciton dissociation (*η*_diss_) and charge collection efficiency (*η*_coll_) could be calculated from the following functions: *η*_diss_ = *J*_ph_ (under short‐circuit condition)/*J*_sat_; *η*_coll_ = *J*_ph_ (under maximum power output condition)/*J*_sat_. The *η*_diss_ is calculated to be 97.43% and 99.45%, and 96.94% for the devices based on PM6/monomer, PM6/**2BTP‐2F‐T,** and PM6/PYF‐T‐*o*, respectively, and their corresponding *η*_coll_ is 85.01%, 91.23%, and 83.15%, respectively. The highest value of *η*_diss_ and *η*_coll_ demonstrates that the **2BTP‐2F‐T‐**based device wins the most efficient charge generation and the best charge collection compared to those of monomer‐ and polymer‐based devices.

The power index *α* (fitted from the function of *J ∝* (*P*_light_)*^*α*^*; *P*_light_ represents the incident light intensity) and the ideal factor *n* (fitted from the function of *V*_oc_ *∝* *n* *kT/q* ln(*P*_light_); *k* is Boltzmann constant, *T* is the temperature in Kelvin, and *q* is the elementary charge) were applied to estimate the recombination mechanism for the three devices. For the device based on PM6/**2BTP‐2F‐T**, *α* is equal to 1 (Figure S5, Supporting Information), indicating that PM6/**2BTP‐2F‐T** is almost free from bimolecular recombination, and the other two systems also show negligible bimolecular recombination with an *α* value of 0.99. What is more, the lowest *n* value of PM6/**2BTP‐2F‐T** (Figure 3f, *n* = 1.15 for PM6/monomer, 1.08 for PM6/**2BTP‐2F‐T**, and 1.26 for PM6/PYF‐T‐*o*), demonstrates that the device based on PM6/**2BTP‐2F‐T** effectively inhibits the trap‐assisted recombination. The decreased *n* value is probably due to its smallest energetic disorder as deduced from its smallest Urbach energy (*E*_U_) value, which is described in the following parts.^[^ ^28^, ^29^ ^]^ The photon‐physical result suggested that the N‐*π*‐N type oligomeric acceptor **2BTP‐2F‐T** could effectively enhance the efficiency of charge generation and charge collection while suppressing trap‐assisted recombination simultaneously. Thus, higher *J*_sc_ and FF are obtained.

### 2.3 Molecular Packing, Morphology, and Charge Properties

**Figure 4:**
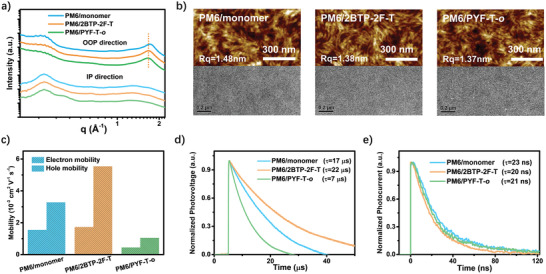
Morphology properties and charge properties: a) 1D GIWAXS curves of optimized blends; b) AFM height images and TEM images for the optimized blends; c–e) charge transport, TPV, and TPC curves for the optimized blend.

The molecular packing and morphology in the optimized blend were further used to confirm the efficient charge generation and collection in the PM6/**2BTP‐2F‐T** blends. As shown in 2D GIWAXS (Figure S6, Supporting Information) and corresponding 1D curves (**Figure** **4**a), all the blends reserved a preferential face‐on packing mode of pristine films; in view of the lamellar peak of PM6, the (010) peak in OOP direction is a combination of the donor and acceptor; therefore, the (100) peak in IP direction could be applied to separate their crystalline ability in the blends. As deduced from the CCL of (100) peak, PM6 exhibited decreased packing ability for PM6/monomer (112 Å), PM6/**2BTP‐2F‐T** (95 Å), and PM6/PYF‐T‐*o* (93 Å), respectively. This trend is totally opposite to the change in their miscibility, which is probably due to the different diffusion speeds of their corresponding acceptors escaping from their mixed regions originating from their differed MW. Consequently, although PM6/**2BTP‐2F‐T** shows smaller miscibility (*χ *= 0.14) than that of PM6/monomer (*χ *= 0.08), however, it forms a more interpenetrating film than that of PM6/monomer, which could be intuitively seen in height atomic force microscopy (AFM) images (Figure 4b) and confirmed by its slightly decreased root‐mean‐square (RMS) surface roughness value (RMS = 1.38 nm). The opposite is also true for the PM6/**2BTP‐2F‐T** and PM6/PYF‐T‐*o*.

The above results indicate that PM6/**2BTP‐2F‐T** blends present an easier morphology control than PM6/monomer and PM6/PYF‐T‐*o*. Furthermore, as shown in the transmission electron microscope (TEM) images (Figure 4b), compared to PM6**/**monomer, more evident fibrous structures scatter in PM6/**2BTP‐2F‐T** blend. Hence, a favorable hole transport route comprising a more interpenetrating network and fibrous structure in PM6/**2BTP‐2F‐T** blend should be responsible for its higher hole mobility (5.54 × 10^−3^ cm^2^V^−1^s^−1^, Figure 4c) than that of PM6/monomer (3.28 × 10^−3^ cm^2^V^−1^s^−1^). The CCLs of (010) peak in the OOP direction for the three blends are at ≈20 Å, and hence, the smallest hole mobility for PM6/PYF‐T‐*o* should be due to its largest *π*–*π* stacking distance in the charge transport direction (3.77 Å) than that of PM6/**2BTP‐2F‐T** (3.73 Å) and PM6/monomer (3.65 Å).

Compared to the *μ*_e_ in their pristine film, the blends exhibited almost no reduced values and the same changing trend, and they are 1.55 × 10^−3^, 1.73 × 10^−3^, and 4.46 × 10^−4^cm^2^ V^−1^ s^−1^ for PM6/monomer, PM6/**2BTP‐2F‐T**, and PM6/PYF‐T‐*o*, respectively. The highest *μ*_e_ with a moderate *π*–*π* stacking further convinced the high morphology tolerance of **2BTP‐2F‐T** and its advantage in morphology regulation. Obviously, the excellent electron/hole transport **2BTP‐2F‐T** in the blend is the combined result of its intra‐molecular charge transport and its well‐ and easier‐tuned morphology featured with fibrous structure, moderate *π*–*π* stacking, and the optimal interpenetrating network. We reiterate that the high tolerance of *μ*_e_ to the morphology and easier‐tuned morphology for N‐*π*‐N oligomeric acceptor are its prominent superiority in OSCs, which potentially push it to obtain the maximized PCE in OSCs.

Besides the best charge transport ability, the optimized morphology for PM6/**2BTP‐2F‐T** is also responsible for its longest charge lifetime of 22 µs, which is much longer than that of PM6/monomer (17 µs) and PM6/PYF‐T‐*o* (7 µs), as characterized by transient photovoltage (TPV, Figure 4d). The results of transient photocurrent (TPC, Figure 4e) also demonstrate that PM6/**2BTP‐2F‐T** exhibits the fastest charge extraction with a lifetime of (19.9 ns), as compared to that of PM6/monomer (23.3 ns) and PM6/PYF‐T‐*o* (21.4 ns). The excellent charge properties for PM6/**2BTP‐2F‐T** facilitate its charge collection and ensure its much higher FF and improved absorbed photon utilization (*J*_sc_).

### 2.4 Energy Loss and Stability

#### 2.4.1 Energy Loss

Energy loss (Δ*E*) measurements were carried out to elucidate the potential of the N‐*π*‐N type oligomeric acceptors. The total Δ*E* can be spitted into three parts: 1) Δ*E*_1_ represents radiative recombination loss above the bandgap, which is based on the Shockley–Queisser (SQ) theory limits. Δ*E*_1_ is directly decided by the energy bandgap. 2) Δ*E*_2_ represents radiative recombination loss below the bandgap; 3) Δ*E*_3_ represents non‐radiative recombination loss below the bandgap, which could be quantified by external quantum electroluminescence efficiency (EQE_EL_).

**Figure 5:**
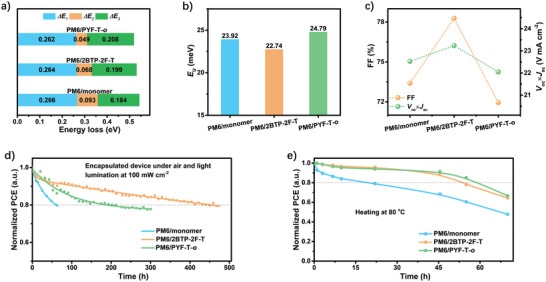
Energy loss and stabilities: a) Energy loss for the three optimized devices; b)Urbach energy; c) improvements of FF and (*V*_oc_ × *J*_sc_) for PM6/**2BTP‐2F‐T**; d,e) stability to light soaking and heating for the three devices fitted by a double exponential decay function.

As shown in **Figure** **5**a and Table S5, Supporting Information, the Δ*E* for the three devices has a very small difference within a value of 0.01–0.02 eV, and the value of PM6/**2BTP‐2F‐T** (0.53 eV) is in between that of PM6/monomer (0.54 eV) and PM6/PYF‐T‐*o* (0.52 eV). The energy band‐gap (*E*_g_) is calculated from the precise measurement of the EQE, with a value of 1.48, 1.45, and 1.42 eV for monomer, **2BTP‐2F‐T,** and PYF‐T‐*o* blend (Figure S7, Supporting Information). Hence, their *V*_oc_ differences mainly result from their different *E*_g_ rather than their Δ*E*. Interestingly, there is a delicate trade‐off between Δ*E*_2_ and Δ*E*_3_ in the three devices. That is, the PM6/monomer‐based device obtains the largest Δ*E*_2_ (0.093 eV), while the smallest Δ*E*_3_ (0.184 eV) and PM6/PYF‐T‐*o* exhibits exactly the reverse result with an Δ*E*_2_ of 0.049 eV and an Δ*E*_3_ of 0.208 eV. As for PM6/**2BTP‐2F‐T**, both the values of Δ*E*_2_ (0.068 eV) and Δ*E*_3_ (0.199 eV) are in between. This is probably because their small energy offset between *E*_g_ and energy of charge transfer (CT) states estimated from normalized fourier‐transform photocurrent spectroscopy external quantum efficiency (FTPS‐EQE) curves and electroluminescence (EL) spectra of devices (as shown in Figure S8 and Table S5, Supporting Information) creates an equilibrium state between the CT states and acceptor singlet, which is believed to enhance the efficiency of radiative decay, while suppressing its non‐radiative decay.^[^ ^30^ ^]^ Hence, the previous analysis indicates there is negligible difference in the Δ*E* for the three systems, and their disparity is due to their slightly different energy levels of themselves and complex energy alignments due to the morphology optimization. Further applying a more rigid molecular backbone or monomer, and proper HOMO energy level of the donor would be a potential approach to decrease the Δ*E* of N‐*π*‐N oligemic type OSCs.

The low degree of energetic disorder at interfaces in the active layers enables free charge separation to outcompete trap‐assisted non‐radiative recombination of the CT state, gaining excellent device performance with small Δ*E*.^[^ ^28^, ^29^, ^31^ ^]^ Hence, Urbach energy (*E*_U_) is obtained through an exponential fit to the FTPS‐EQE spectra to evaluate the degree of energetic disorder (Figure 5b and Figure S9, Supporting Information).^[^ ^32^ ^]^ The PM6/**2BTP‐2F‐T** obtains the smallest *E*_U_ with a value of 22.74 meV, compared to that of PM6/monomer (*E*_U_ = 23.92 meV) and PM6/ PYF‐T‐*o* (*E*_U_ = 24.79 meV), indicating the smallest width of the tail of the electronic density of disorder (DOS). It should be mentioned that all the *E*_U_ for the three devices is comparable to the thermal energy *k*_B_ *T* (25.8 meV) at room temperature. The small *E*_U_ for the three devices guarantees their minimized loss of (Δ*E*_2_ + Δ*E*_3_) (<0.28 eV). The smallest *E*_U_ of PM6/**2BTP‐2F‐T** enables less trap‐assisted recombination of the charge, and a much longer charge lifetime, benefiting the improvements of FF and *J*_sc_.

Hence, PM6/**2BTP‐2F‐T** exhibits more efficient charge generation, higher absorption coefficient, and an optimal morphology with a smaller energetic disorder, leading to ultimately a high *J*_sc._ The much higher *J*_sc_ with similar Δ*E* for PM6/**2BTP‐2F‐T** leads to a maximized (*V*_oc_ × *J_s_*_c_) in the three devices (Figure 5c), which is a bottleneck for the current photovoltaic materials design and device optimization. Together with excellent charge properties, it wins the champion PCE in the three systems. Furthermore, its small *E*_U_, high tolerance of *μ*_e_ to the morphology, and easily tuned morphology indicate a potential of low Δ*E*, efficient charge generation, and excellent charge transport properties. If a more matched polymer in energy level could be found, further improved device performances in OSCs would be expected based on N‐*π*‐N oligomeric acceptors.

#### 2.4.2 Stability

The much higher MW of **2BTP‐2F‐T** than monomer would lead to a lower relative diffusion ability and better stability, as proved in polymer acceptors.^[^ ^15^ ^]^ The T_80_ (sustained 80% of the initial efficiency) of the three encapsulated devices to light soaking (100 mW cm^−2^) under air conditions and thermal stability at 80 °C under a nitrogen atmosphere were investigated. Conventional devices with ITO/poly(3,4‐ethylenedioxythiophene):poly(styrenesulfonate) (PEDOT:PSS)/active layer/PNDIT‐F3N/Ag structure were used in the light‐soaking stability and thermal stability test. Figures 5d and 5e show the trend of PCE decay over time in light‐soaking stability and thermal stability test, respectively. The curves in Figure 5d were fitted well by a double exponential decay function to obtain a relatively accurate T_80_, while curves in Figure 5e were obtained by the b‐spline algorithm.

Expectedly, the lifetime of T_80_ to light soaking under air condition based on PM6/**2BTP‐2F‐T** device exhibits 7 times longer (443 h) than that of PM6/monomer (62 h); beyond belief, it also demonstrates 2.4 times longer than that of PM6/PYF‐T‐*o* (185 h). Besides the morphology factors, the higher optical chemical stability of the **2BTP‐2F‐T** was also observed in a recently reported result measured under a nitrogen atmosphere.^[^ ^33^ ^]^ The lifetime of T_80_ to heat treatment for the PM6/**2BTP‐2F‐T** is also much longer than that of PM6/monomer (54 h vs 20 h) and comparable to that of all‐polymer OSCs PM6/PYF‐T‐*o* (57 h). Noteworthy, further prolonging the heating time, the PM6/**2BTP‐2F‐T** shows a slightly decreased rate of device attenuation than that of PM6/PYF‐T‐*o* (Figure 5e). The above results demonstrate that PM6/**2BTP‐2F‐T** possesses comparable thermal stability but better light‐soaking stability than PM6/PYF‐T‐*o*, and is much better than PM6/monomer. This difference in stability was further convinced by the less variated morphology of PM6/**2BTP‐2F‐T** films under the heating and light‐soaking process, as shown in Figure S10, Supporting Information.

## 3 Conclusion

In this article, we designed an N‐*π*‐N type oligomeric acceptor **2BTP‐2F‐T**. Using its corresponding monomer and polymer as references, we profoundly demonstrated the advantages of **2BTP‐2F‐T**, including a higher absorption coefficient, improved electron mobility with less dependence on molecular packing, and easier morphology control with polymer donors. Using PM6 as the donor, the PM6/**2BTP‐2F‐T** blend exhibits superior advantages compared with their monomeric and polymeric counterparts: 1) More favorable morphology, including the smallest energetic disorder, moderate *π*–*π* stacking, and appropriate interpenetrating network; 2) Improved charge properties, including higher hole/electron mobility, longer charge lifetime, and faster charge extraction; 3) Efficient charge generation with similar energy loss; 4) Better light‐soaking stability and comparable thermal stability to its polymeric counterparts. The high efficiency of over 18% in OSCs, together with its improved stability, suggests a bright future for the N‐*π*‐N oligomeric acceptor type OSCs.

## Conflict of Interest

The authors declare no conflict of interest.

## Supporting information

Supporting Information

## Data Availability

The data that support the findings of this study are available from the corresponding author upon reasonable request.
