# Supplementary material for: “N‐π‐N” Type Oligomeric Acceptor Achieves an OPV Efficiency of 18.19% with Low Energy Loss and Excellent Stability
Source: Adv Sci (Weinh). 2022 Jun 16;9(23):2202513. doi: 10.1002/advs.202202513 (PMC9376851; doi:10.1002/advs.202202513)
Supplement: Supplementary file 1 — Supporting Information [file ADVS-9-2202513-s001.pdf]

Supplementary Information

New type of "N-π-N" oligomeric acceptor enables an efficiency of 18.19% with excellent stability and low energy loss

Lili Zhang#, Ziqi Zhang#, Dan Deng, Huiqiong Zhou, Jianqi Zhang, and Zhixiang Wei**

1. Synthesis Methods

Monomer: BTP-CHO (700 mg, 1.0eq), two kind of terminal groups IC-2F(154.4 mg, 1.2eq) and IC-Br (183.2 mg, 1.2eq) were added to a three-necked flask with 50 mL CF, the reaction mixture was first stirred at R.T. for 3h, then heated to 65 ℃ and stirred overnight. After cooling to room temperature, the reaction mixture was poured into water and extracted with DCM for three times. The crude product was further purified by silica gel column chromatography (petroleum ether: DCM= 3:1) to afford monomer as dark blue solid. (399 mg, 41.5%). ^{1}H NMR (400 MHz, CDCl3) δ 9.18 (d, J = 8.8 Hz, 2H), 8.58 (d, J = 8.4 Hz, 2H), 8.04 (d, J = 1.9 Hz, 1H), 7.87 (dd, J = 8.4, 1.9 Hz, 1H), 7.70 (t, J = 7.5 Hz, 1H), 4.76 (d, J = 7.5 Hz, 4H), 3.24 (t, J = 7.7 Hz, 4H), 2.10 (d, J = 5.7 Hz, 2H), 1.96 – 1.78 (m, 4H), 1.38 (s, 4H), 1.26 (s, 26H), 1.19 – 1.08 (m, 12H), 1.00 (s, 30H), 0.83 (dt, J = 14.4, 6.8 Hz, 20H), 0.68 (t, J = 5.7 Hz, 6H).

2BTP-2F-T: AS-BTP (251.8 mg, 3eq) and T-Sn(20 mg, 1eq) were added into Schlenk tube with toluene under the nitrogen atmosphere, after vacuuming and refilling with argon for three times, Pd2(dba)3 and P-(o-toyl) were added to the reaction flask and continue purging with N2 for 10 mins. The reaction was heated to 110°C and stirred for another 48h. After cooling down, the reaction mixture was participated into methanol. The solid was filtered and further purified with silica gel column chromatography (petroleum ether: $CHCl_3$ = 1:3) to afford the dimer as a black solid (116 mg, 70.7%).^{1}H NMR (400 MHz, CDCl3) δ 9.16 (s, 2H), 9.13 (s, 2H), 8.76 (d, J = 8.3 Hz, 2H), 8.57 (dd, J = 9.8, 6.5 Hz, 2H), 8.12 (s, 2H), 7.99 (d, J = 9.1 Hz, 2H), 7.69 – 7.60 (m, 4H), 4.81 (s, 8H), 3.22 (d, J = 7.1 Hz, 6H), 2.17 (s, 4H), 1.88 (dd, J =

14.7, 7.4 Hz, 8H), 1.30 (t, J = 25.0 Hz, 74H), 1.02 (s, 70H), 0.87 (t, J = 6.6 Hz, 20H), 0.78 (dt, J = 9.7, 7.2 Hz, 16H), 0.71 (dd, J = 11.4, 6.7 Hz, 12H). MALDI-TOF MS (m/z):3358.650.

2. Molecular properties characterization

The UV-Vis spectra for solution and films were obtained by Perkin Elmer Lambda950 spectrophotometer. The square wave voltammetry (SWV) and CV (cyclic voltammetry) measurement was carried out by an electrochemical workstation (CHI660E) with glassy carbon electrode coated with films, Pt plate, and Ag/Ag^{+} electrode acting as the working, counter, and reference electrodes, respectively, in a 0.1 mol L^{-1} tetrabutylammonium phosphorus hexafluoride (Bu_4NPF_6) acetonitrile solution. Redox potential of Ag/Ag^{+} electrode was calibrated using the ferrocene/ferrocenium (Fc/Fc^{+}) redox couple (−4.8 eV) by CV measurement. The optimized molecular structure was calculated by density functional theory (DFT) method at the B3LYP/6-31G(d,p) level, with software Gaussian 09.

Contact angle (θ) measurements were conducted through DSA 100 with deionized water and glycerol. The samples were prepared by casting the solutions onto the ITO/glass substrate.

According to Owens-Wendt method, surface energy could be divided into dispersive and polar components.

$$\gamma = \gamma^{d} + \gamma^{p} \quad (1)$$

Furthermore, the dispersive and polar surface energy can be calculated based on the equation below:

$$(1 + \cos\theta)\,\gamma_L = 2\sqrt{\gamma_S^{d}\gamma_L^{d}} + 2\sqrt{\gamma_S^{p}\gamma_L^{p}} \quad (2)$$

where θ is the contact angle of a specific solvent, γ_L is the surface energy of the solvent, γ_S^{d} and γ_S^{p} refer to the dispersive and polar surface energy of the solid, respectively, and γ_L^{d} and γ_L^{p} refer to the dispersive and polar surface energy of the

solvent, respectively.

Thus, the unknown value γ_S^d and γ_S^p can be obtained though combining two equations using contact angles of two different solvents.

Solubility parameter (δ) can be calculated from the surface energy,

$$\delta = K\sqrt{\gamma} \quad (3)$$

where γ is the surface energy, K is the proportionality constant (K = 116 × 10^3 $m^{-1/2}$).

And Flory–Huggins interaction parameter (χ_{ij}) can be written as a function of two solubility parameter,

$$\chi_{ij} = \frac{V_0}{RT}(\delta_i - \delta_j)^2 \quad (4)$$

where χ_{ij} is the Flory–Huggins interaction parameter between the material i and j, V_0 is the geometric mean of the polymer segment molar volume, R is the gas constant, T is the absolute temperature, and δ_i and δ_j are the solubility parameter of material i and j, respectively.

To simplify, we define the parameter $\kappa = K^2V_0/RT$, then the Flory–Huggins interaction parameter can be written as the formula below,

$$\chi_{ij} = \kappa(\sqrt{\gamma_i} - \sqrt{\gamma_j})^2 \quad (5)$$

where γ_i and γ_j are the surface energy of material i and j, respectively.

3. Devices fabrication and characterization

The devices were fabricated with a conventional structure of ITO/2PACz/active layer/PNDIT-F3N/Ag. The ITO-coated glass substrates were washed with detergent, deionized water, alcohol, and isopropanol in an ultrasonic bath for 30 minutes sequentially. Then the washed ITO glass was treated with UV-ozone for 15 minutes. 2PACz was spin-coated onto ITO for 30 seconds and then annealed at 100°C for 10

minutes. Donor and acceptor materials were dissolved in chloroform (CF) solvent with a total concentration of 14 mg/ml (donor: acceptor = 1:1.2 w/w) and then stirred at 50 ℃ for 60 minutes. 1-Chloronaphthalene (CN) was then added into the solvent at the volume fraction of 2% for PM6/PYF-T-o solvent and 1% for the other two solvents. After another 30 minutes of stirring, the blend solution was spin-coated at 3000 rpm for 30 seconds to form a thin film on the substrate. Thermal annealing at 100 ℃ for 10 minutes was utilized to optimize the morphology of the active layer. Then, PNDIT-F3N was spin-coated onto the top of the active layer as an electron transport layer at 2000 rpm for 30 seconds. Finally, 160 nm of Ag was deposited onto the active layer to form a back electrode.

One solar cell device consists of four cells, and the active area of each cell is approximately 4 mm^2. The whole photovoltaic performance characterization was processed in an N_2-filled glovebox with a 2.56 mm^2 mask. The *J*−*V* measurements were performed via the solar simulator (SS-F5-3A, Enlitech) along with AM 1.5 G spectra, whose intensity was calibrated by the certified standard silicon solar cell (SRC-2020, Enlitech) at 100 mW cm^{-2}. *J*–*V* measurement signals were recorded by Keithley 2400 source-measure unit. The EQE spectrum was measured through the Solar Cell Spectral Response Measurement System FETOS-QE-3011 (Enli Technology Co., Ltd., Taiwan).

Transient photocurrent (TPC) and photovoltage (TPV) were measured by applying a 488 nm solid-state laser (Coherent OBIS CORE 488LS) with a pulse width of ~30 ns. The current traces were recorded by a mixed domain oscilloscope (Tektronix MDO3032) through converting the registered voltage drop across a 2 Ω resistor load connected in series to the solar cell. TPV measurements were performed on the solar cell at the open-circuit condition with the same pulsed laser. The photovoltage traces were registered by the oscilloscope with an external 10 MΩ resistor in series.

4. Charge carrier mobility characterization

Hole and electron mobilities were measured by the space-charge limited current (SCLC) method with hole-only devices and electron-only devices. The hole-only devices adopted ITO/PEDOT:PSS/active layer/MoO_x/Ag structure, while electron-only devices adopting ITO/ZnO/active layer/PNDIT-F3N/Ag structure. The active layers for these two devices were spin-coated under the same condition as that of solar cells. *J–V* curves in the range of 0 to 5 V were gained by Keithley 2400 source-measure unit in the dark condition.

The mobilities were obtained by fitting *J-V* curves with the formula of:

$$J = \frac{9\varepsilon_0\varepsilon_r\mu V^2}{8L^3}\exp(0.89\beta\sqrt{\frac{V}{L}}) \quad (6)$$

where J is the current density, L is the thickness of the active layer, μ is the mobility, ε_0 is the vacuum dielectric constant, ε_r is the relative dielectric constant of the transport medium, V (= V_{app} - V_{bi}) is the internal voltage, where V_{app} is the applied voltage and V_{bi} is the built-in voltage.

5. Morphology and crystallization characterization

Transmission electron microscopy (TEM) characterization was performed by Tecnai G2 F20 U-TWIN TEM instrument. AFM measurements were performed on Multimode 8 with ScanAsyst mode. Grazing incidence wide angle x-ray scattering (GIWAXS) measurement was conducted at XEUSS SAXS/WAXS equipment.

6. Energy loss characterization

Fourier-transform photocurrent spectroscopy external quantum efficiency (FTPS-EQE) was measured by using an integrated system (PECT-600, Enlitech). Electroluminescence quantum efficiency (EQE_{EL}) measurements were performed by applying external voltage/current sources through the devices (REPS, Enlitech).

7. Statistical Analysis

The data was direct output from the software that comes with the test equipment and then pre-processing with a simple evaluation of outliers. For TPC, TPV, and stability test, physical quantities including current, voltage, and PCE were normalized to express their relationship against time. Statistical analysis was performed by the average and standard deviation from devices in the same condition, which was expressed as mean ± SD. The sample size for statistical analysis was described in the corresponding table and expressed as n. The average and standard deviation were calculated by the Statistical Functions in Microsoft Excel. Data analysis and graphing were performed by Microsoft Excel and Origin.

8. Supplementary Scheme and Figures

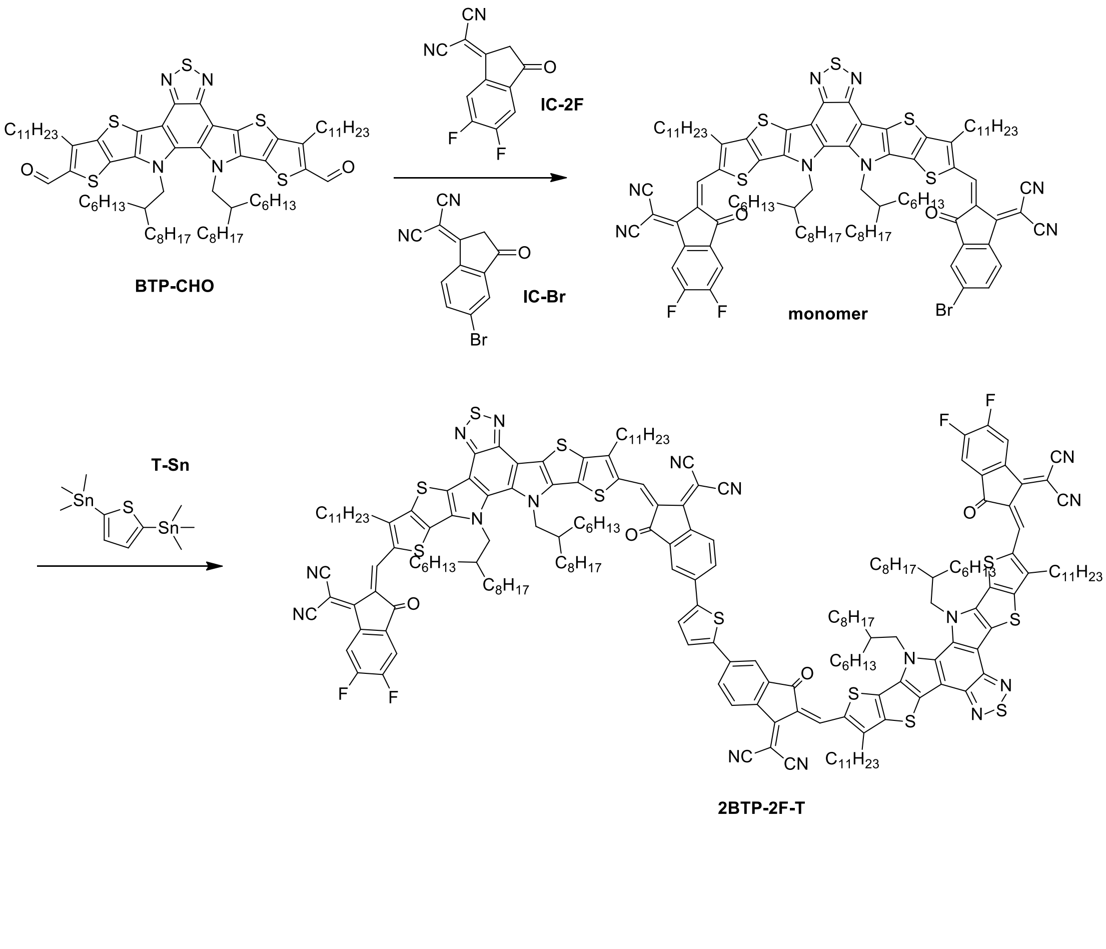

Scheme 1 Synthesis procedure of dimer 2BTP-2F-T.

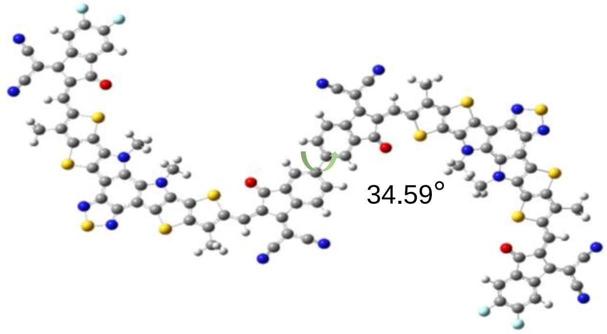

Figure S1. Rotation angle of the monomer directly linked.

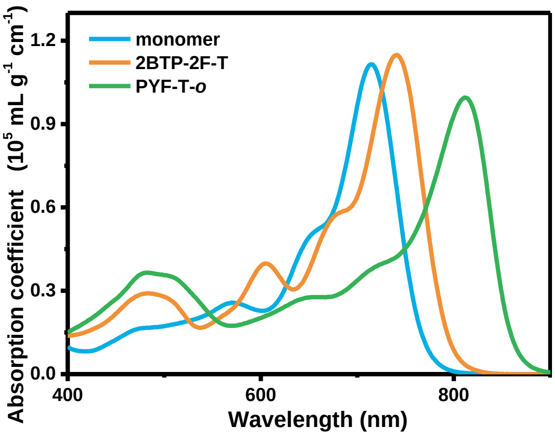

Figure S2. Absorption coefficient in solution

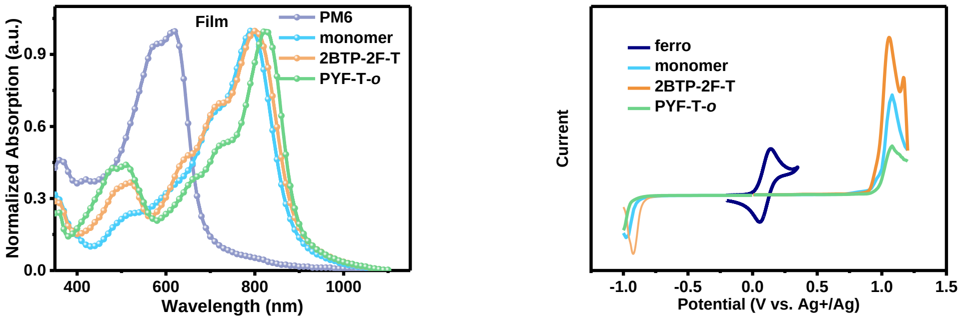

Figure S3. a) Normalized Absorption spectrum of the three acceptors in the film; b) SWV curves for three acceptors and CV curve for ferro.

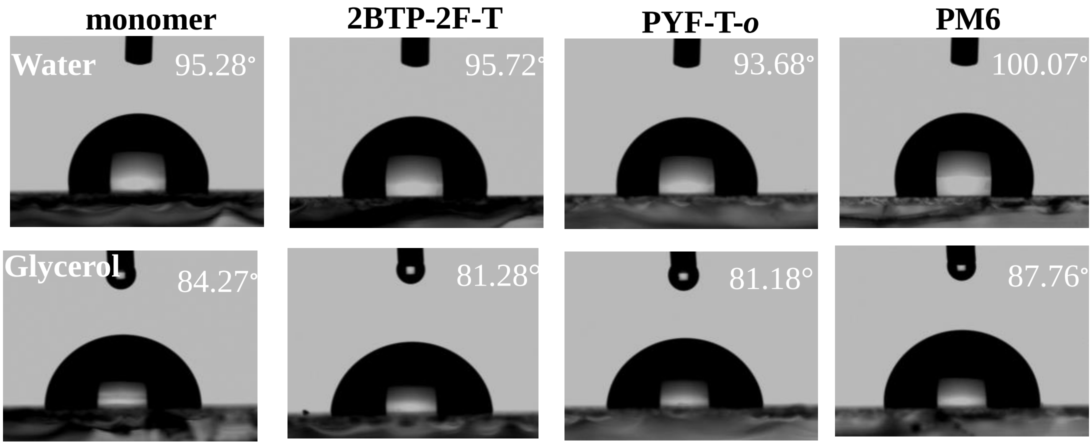

Figure S4. Contact angles of three acceptors and PM6

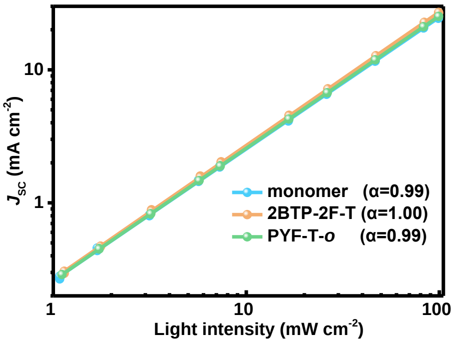

Figure S5. J_{sc} dependence on incident light intensity.

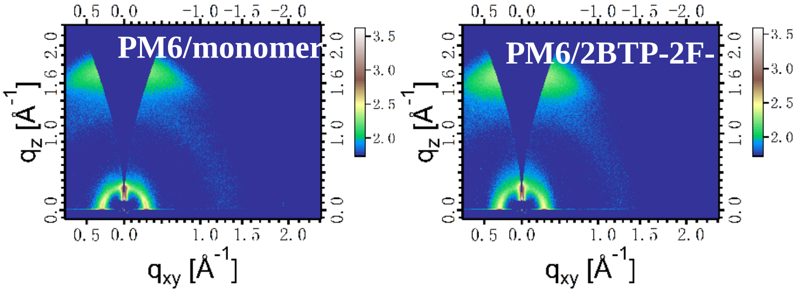

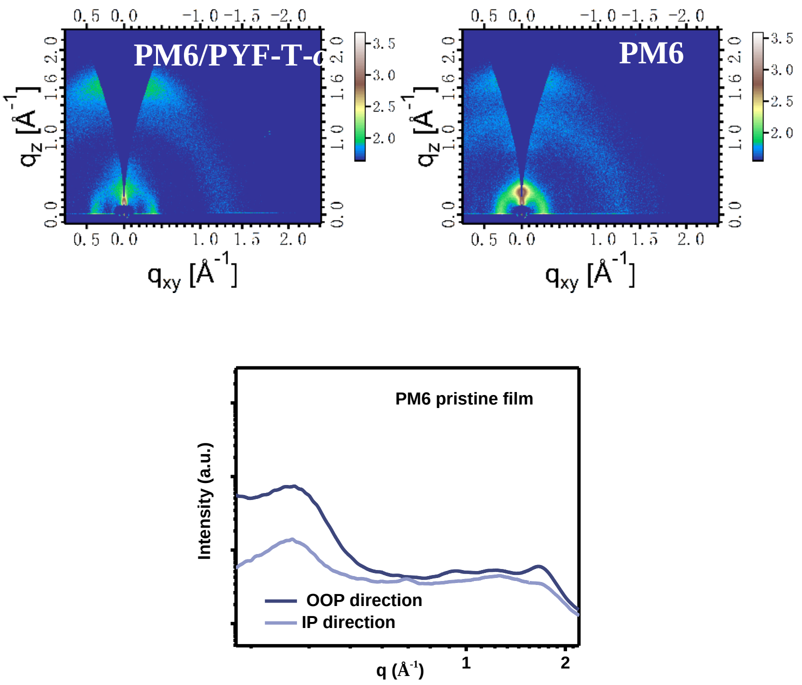

Figure S6. GIWAXS images for blends and PM6 pristine film.

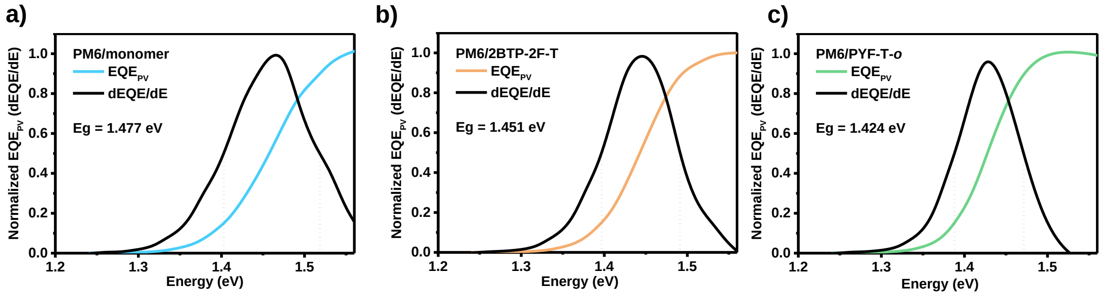

Figure S7. a-c) Energy band-gap measured from the EQE.

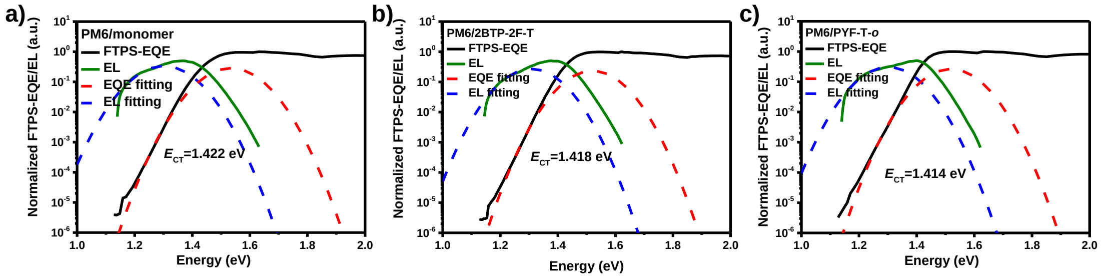

Figure S8. a-c)Normalized FTPS-EQE curves EL spectra and fitting of the charge transfer state energy.

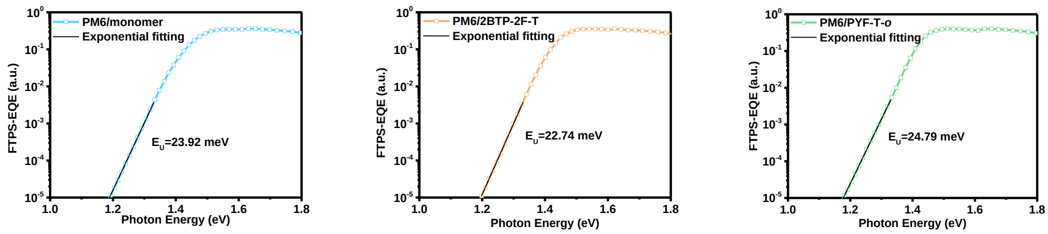

Figure S9. FTPS-EQE curves and analysis of the energetic disorder.

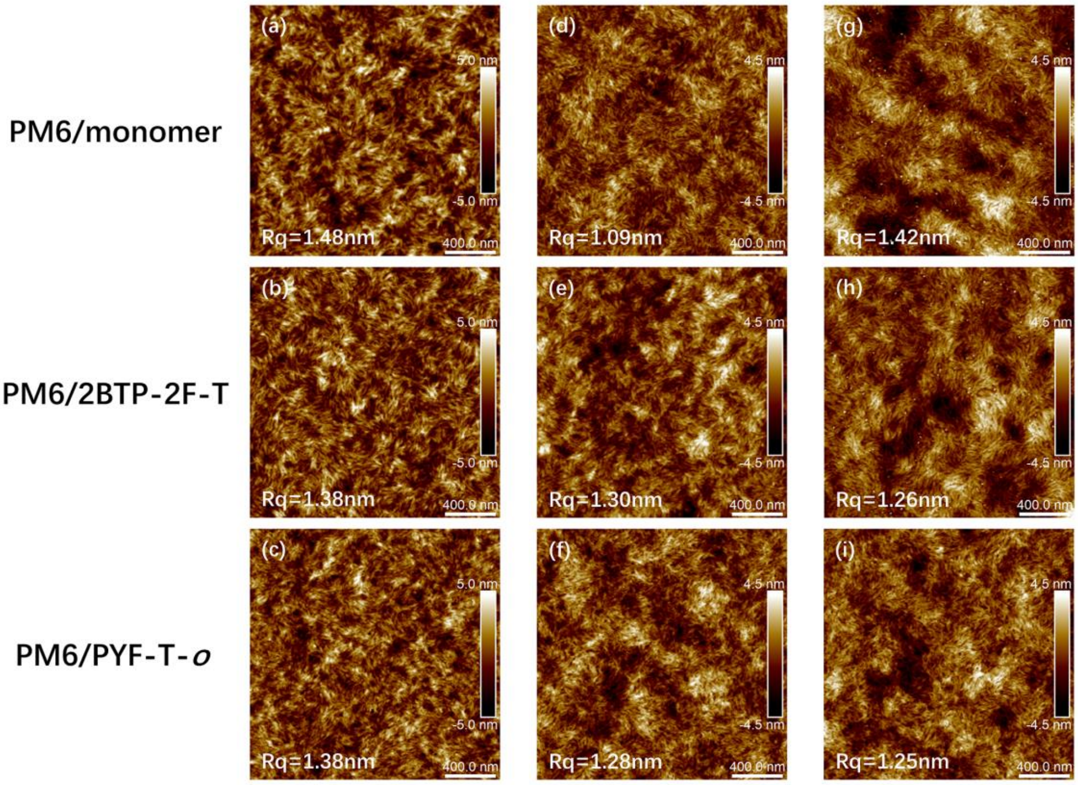

Figure S10. AFM height images of PM6/monomer, PM6/2BTP-2F-T, and PM6/PYF-T-o films without treatment (a-c), after heating (d-f), and after the light-soaking process (g-i), respectively.

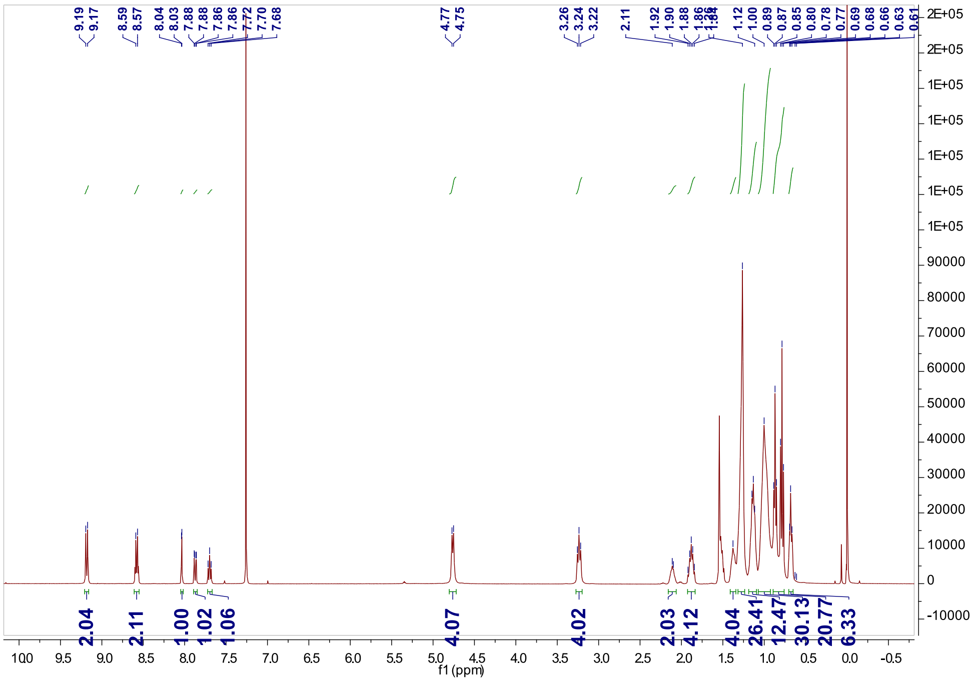

Figure S11. ^{1}H NMR spectrum of monomer in solution

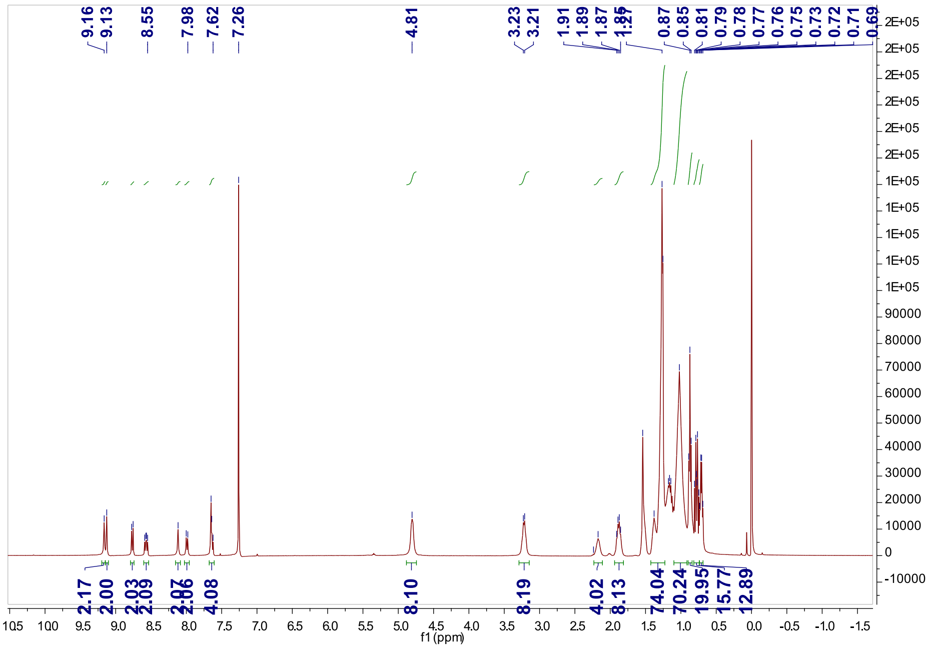

Figure S12. ^{1}H NMR spectrum of 2BTP-2F-T in solution

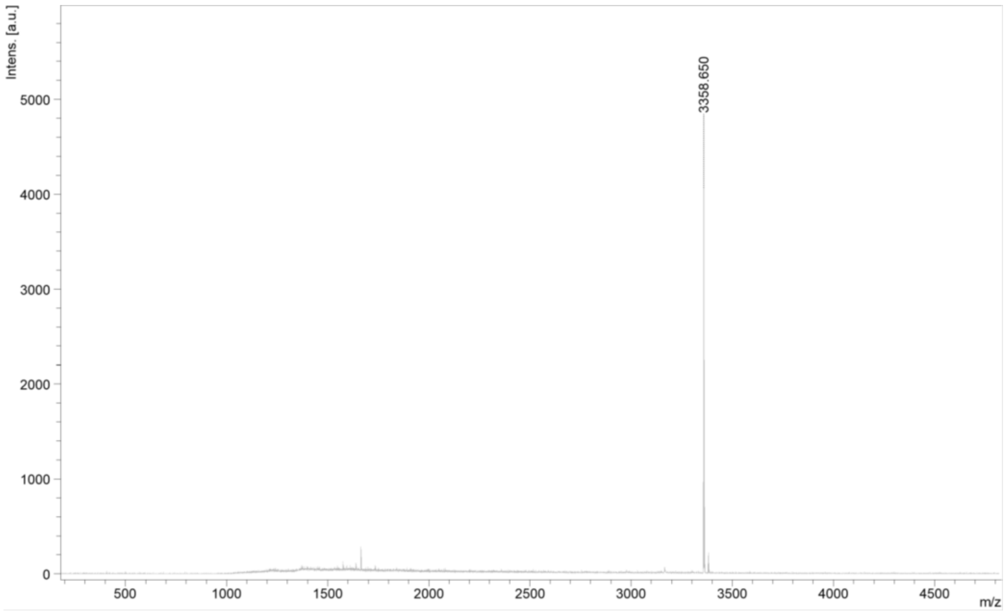

Figure S13. MALDI-TOF of 2BTP-2F-T

9. Supplementary Tabless

Table S1 The detailed optoelectronic parameters of three acceptors.

	$\lambda_{max,sol}$ [nm]	$\lambda_{max,film}$ [nm]	$\lambda_{onset,film}$ [nm]	$\alpha_{max,film}$ [$10^5 cm^{-1}$]	E_g [eV][a]	HOMO [eV][b]	LUMO [eV][b]	E_g [eV][b]
**monomer**	714	792	885	1.23	1.40	-5.68	-3.81	1.87
**2BTP-2F-T**	740	801	892	1.33	1.39	-5.67	-3.84	1.83
**PYF-T-*o***	811	824	895	1.19	1.39	-5.64	-3.82	1.82

[a] the optical gap calculated from the absorption onset of films by the equation: E_g= 1240/λ (eV).

[b] the energy levels and energy gap calculated from SWV.

Table S2 Device optimization of thermal annealing (TA) temperature for PM6/2BTP-2F-T. The average values and standard deviations were obtained from 6 devices, which were expressed as mean ± SD, n = 6.

TA temperature	V_{oc} [V]	J_{sc} [mA cm^{-2}]	FF [%]	PCE [%]
**80 °C**	0.910	23.04	78.23	16.41 (16.11 ± 0.31)
**90 °C**	0.902	23.60	78.76	16.76 (16.50 ± 0.37)
**100 °C**	0.903	23.84	78.63	16.93 (16.68 ± 0.20)
**110 °C**	0.889	24.72	73.26	16.10 (15.78 ± 0.30)
**120 °C**	0.883	24.05	74.92	15.90 (15.46 ± 0.36)

Table S3 Device optimization of CN additive ratio for PM6/2BTP-2F-T. The average values and standard deviations were obtained from 6 devices, which were expressed as mean ± SD, n = 6.

The ratio of CN additive	V_{oc} [V]	J_{sc} [mA cm^{-2}]	FF [%]	PCE [%]
**0.5%**	0.906	24.79	74.81	16.81 (16.61 ± 0.22)
**1%**	0.902	24.41	78.47	17.27 (17.12 ± 0.08)
**1.5%**	0.901	24.01	77.84	16.84 (16.63 ± 0.15)

**2%**	0.907	23.16	78.69	16.53 (16.39 ± 0.08)

Table S4 Device optimization of different hole transport layer (HTL) and electron transport layer (ETL) for PM6/2BTP-2F-T. The average values and standard deviations were obtained from 6 devices, which was expressed as mean ± SD, n = 6.

**HTL & ETL**	**V_{oc} [V]**	**J_{sc} [mA cm^{-2}]**	**FF [%]**	**PCE [%]**
**PEDOT&PFNBr**	0.903	24.49	78.49	17.37 (17.28 ± 0.09)
**PEDOT&F3N**	0.903	24.66	78.19	17.41 (17.30 ± 0.12)
**2PACz&PFNBr**	0.897	25.04	79.05	17.74 (17.61 ± 0.12)
**2PACz&F3N**	0.911	25.50	78.28	18.19 (18.06 ± 0.11)

Table S5 The detailed parameters of energy loss for three devices

**Acceptor**	**V_{oc} [V]**[a]	**E_g [eV]**	**E_{loss} (E_g-eV_{oc})**	**$\Delta E1$ [eV]**	**$\Delta E2$ [eV]**	**$\Delta E3$ [eV]**	**$E_{loss,cal}$ [eV]**	**$V_{oc,cal}$ [V]**
**monomer**	0.935	1.477	0.542	0.266	0.093	0.184	0.543	0.934
**2BTP-2F-T**	0.917	1.451	0.534	0.264	0.068	0.199	0.531	0.920
**PYF-T-*o***	0.900	1.424	0.524	0.262	0.049	0.208	0.519	0.905

[a] the V_{oc} was measured without mask.
